# Mediation of 10-Year Cardiovascular Disease Risk between Inflammatory Diet and Handgrip Strength: Base on NHANES 2011–2014

**DOI:** 10.3390/nu15040918

**Published:** 2023-02-12

**Authors:** Zechun Xie, Ling Wang, Mengzi Sun, Rui Wang, Jing Li, Xuhan Wang, Ruirui Guo, Yibo Dong, Yuxiang Wang, Bo Li

**Affiliations:** Department of Epidemiology and Biostatistics, School of Public Health, Jilin University, Changchun 130021, China

**Keywords:** inflammatory diet, cardiovascular disease, handgrip strength, NHANES, mediation

## Abstract

Objective: Dietary inflammatory index (DII) and handgrip strength (HGS) were correlated, and both were associated with cardiovascular disease (CVD). However, the role of the 10-year CVD risk in the relationship between DII and grip strength remains uncertain. Methods: This study involved 5691 adults from the National Health and Nutrition Examination Survey (NHANES) in 2011–2014. Dietary inflammation, 10-year CVD risk and relative grip strength were assessed by the Dietary Inflammation Index, the Framingham Risk Score (FRS) and handgrip strength adjusted BMI. Linear regression analyses and mediation analysis were used to explore these associations. Results: Both DII and 10-year CVD risk were negatively associated with relative handgrip strength, and DII was positively associated with 10-year CVD risk. Additionally, 10-year CVD risk partially mediated the association between DII and relative handgrip strength by a 11.8% proportion. Specifically, the mediating effect of the 10-year risk of CVD varied by gender and age. Conclusions: Reducing the 10-year risk of CVD attenuates the effect of an inflammatory diet on relative grip strength impairment. Therefore, we recommend reducing the effect of inflammatory diet on grip strength impairment by controlling any of the FRS parameters, such as lowering blood pressure and smoking cessation, especially with targeted measures for different populations.

## 1. Introduction

The loss of muscle mass and strength is very common, even by more than 50% in the elderly population over 80 years of age, making it a major public health problem in an aging society. The pathogenesis of muscle mass and strength loss is complex, and recent studies suggest that it may be related to inflammation, such that patients with chronic inflammatory diseases, such as rheumatoid arthritis, lose muscle mass and strength more rapidly than healthy subjects [1]. Handgrip strength is a quantification of muscle strength and bone health [1,2] and is the simplest and least costly way to assess muscle mass and function in clinical practice [3]. At the same time, low grip strength is an objective response to the frailty, and when a person is in a weak state, his control over strength is diminished [4]. A meta-analysis has found that the prevalence of low HGS was 10–27% in people over 18 years of age, and the prevalence is lower in men than in women [5]. Low HGS has a high association with health hazards associated with a higher risk of all-cause mortality and CVD death [6]. Thus, HGS is often used as a predictor of death and disability. Therefore, the maintenance and improvement of muscle health has become a public health issue of global concern.

The major cause of death is CVD, which includes atherosclerosis, cerebrovascular disease, and peripheral arterial disease. CVD accounted for 31% of global mortality in 2012 [7], and one-third of deaths in the United States were caused by CVD, creating a heavy medical burden [8]. The inflammatory environment is the basis for CVD production, and the intake of anti-inflammatory diets rich in antioxidants (fruits, vegetables, fiber) can be cardioprotective by increasing adherence to anti-inflammatory foods to reduce low-level systemic inflammation [7]. We know that CVD patients often experience frailty, especially frailty in the limbs, due to reduced physical activity [9]. The 10-year CVD risk (FRS) is a single multivariate risk function developed by Framingham to predict the risk of developing all CVD over the next 10 years [10]. A review including nine studies showed an OR of 2.7–4.1 for CVD versus frailty and an OR of 1.5 for non-frail individuals [11].

Inflammation characterized by elevated blood inflammatory markers is a cause of chronic disease, frailty and premature death. Inflammation affects frailty and CVD mainly by inhibiting growth factors, accelerating catabolism and interfering with homeostatic signaling transmissions [12]. Previous studies have shown that diet is associated with the development of inflammation [13]. The inflammation in the body comes mainly from the diet [14], which leads to various chronic diseases. Inflammation can lead to muscle atrophy by inhibiting muscle fibers [15]. A cross-sectional study in Iranian adults concluded that a higher Dietary Inflammation Index was associated with reduced grip strength [16]. Similar findings emerged from Shivappa’s cohort study that a pro-inflammatory diet led to higher incidence of frailty [17]. A retrospective study demonstrated that diet affects CVD risk not through the action of a single nutrient, but through the interaction of several nutrients with genetic factors. Therefore, it is more important to focus on dietary patterns rather than single nutrients when studying the relationship between diet and CVD risk [18]. The Dietary Inflammation Index (DII) was developed by Shivappa as a tool to assess the potential inflammatory in different foods [19]. A growing number of studies have found that DII is associated with increased risk of CVD with gender and age specificity [20,21].

However, research on the connection between the dietary inflammatory index, 10-year risk of CVD and handgrip strength is scarce. Therefore, in this study we aim to explore whether 10-year risk of CVD mediates the relationship between dietary inflammatory index and handgrip strength and whether it differs by gender and age subgroups.

## 2. Methods

### 2.1. Study Design and Participants

**Figure 1:**
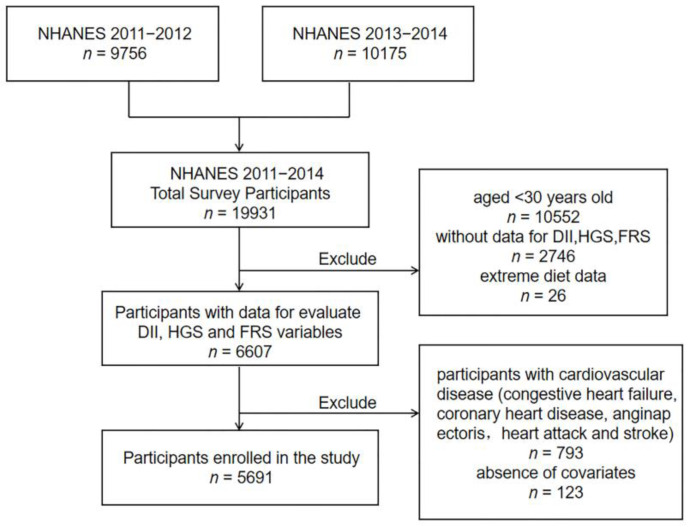
Flowchart for study population selection. Abbreviations: DII, Dietary Inflammatory Index; HGS, handgrip strength; FRS, Framingham Risk Score.

Data for our study were obtained from the National Health and Nutrition Examination Survey (NHANES), a cross-sectional study conducted by the National Center for Disease Prevention and Control (CDC) in which all participants provided written informed consent. NHANES used a complex probability sampling design and consisted of two main components: a household interview and a health check-up [22]. The total number of participants in this survey from 2011–2014 was 19,931, and according to the exclusion criteria, participants younger than 30 years, participants with CVD (congestive heart failure, coronary heart disease, angina, heart attack and stroke), extreme dietary data (total energy intake for women <500 or >5000 kcal/day for women and <500 or >8000 kcal/day for men), and participants with missing information were excluded, resulting in a total of 5691 participants included in this study. The specific study population selection process is shown in Figure 1.

### 2.2. Measurements

#### 2.2.1. Dietary Inflammatory Index (DII)

Dietary data were derived from self-reports, and participants underwent two 24 h recall interviews, the first face-to-face at a mobile examination center and the second by telephone 3–10 days later, with results averaged over the two occasions. Shivappa et al. [19] developed the DII which was used to assess the potential level of inflammation of the diet. Higher DII scores are associated with a more pro-inflammatory diet, and lower DII scores indicate a more anti-inflammatory diet. The DII is calculated using 45 food parameters. These parameters are then used to derive the participant’s exposure relative to the standard global mean as a z-score. Z-cores were obtained by subtracting the daily intake from the mean of a regionally representative database and dividing this value by the standard deviation of the daily intake. To minimize the effect of outliers or positive deviations, the z-score was converted to a percentage (values from 0–1), doubled and subtracted by 1 to obtain a symmetric distribution centered on “0”. Next, the obtained values were multiplied by the corresponding overall food parameter scores, and the scores for the 27 nutrients were summed as the final DII score for each subject as a definition of anti-inflammatory and pro-inflammatory diets with a 0-position cut-off [19].

#### 2.2.2. Cardiovascular Disease Risk

We used the Framingham Risk Score (FRS) to assess participants’ risk of CVD over the next 10 years (10). According to the Framingham Heart Study 10-Year Cardiovascular Risk Calculator, FRS scores were based on sex (male or female), age (years), HDL cholesterol (mg/dL), total cholesterol (mg/dL), diabetes status (yes or no, diabetes defined as fasting glucose ≥126 mg/dL or physician diagnosis of diabetes), smoking status (yes or no, smoking defined as current smokers and nonsmokers as former smokers and never smokers), and treated or untreated (as determined by whether your doctor has ever told you that you have hypertension and whether you take prescribed medication for hypertension) systolic blood pressure. There were separate scales for men and women [10]. The resulting total score is the risk of CVD over 10 years, expressed as FRS%.

#### 2.2.3. Assessment of Handgrip Strength

Subjects’ handgrip strength was assessed using a Takei dynamometer (TKK5401; Takei Scientific Instruments, Tokyo, Japan). Detailed instructions for use are provided in the NHANES Muscle Strength Procedures Manual. Before starting the test, the examiner demonstrated and explained the measurement procedure, adjusted the Takei dynamometer to fit the subject’s hand and performed a simulated measurement. Next, both hands were repeated three times, with a 60 s rest period between each measurement. The measurement was valid when the subject was able to stand and form a 90° angle with the index finger on the dynamometer handle. Because the relationship between muscle strength and body mass is strongly linked [23], cut values for clinically meaningful low muscle mass adjusted for BMI were published by the Foundation for the National Institutes of Health Sarcopenia Project [24]. So, in our study relative grip strength was defined as the mean of dominant hand adjusted for BMI (i.e., HGS/BMI) [25].

#### 2.2.4. Covariate Assessment

Demographic information was self-reported through household interviews. Age was used as a continuous type variable in the control variables and was divided into the following groups in the subgroup analysis: 30–44 years old, 45–64 years old or ≥65 years old [26]. Gender was reported as male or female. Race was categorized as white, black or other races [27]. Marital status was expressed as married, divorced or single [28]. Educational level was divided as less than high school or at least high school level [29]. Height and weight were obtained from physical examination data. Body weight was measured with a Toledo scale, and height was measured with a straightedge. Body mass index (BMI) was obtained as a continuous variable from weight (kg)/height (m^2^). Physical activity was assessed using a questionnaire and divided into two groups: “active” and “inactive”. The active group was defined as those with more than 149 min of moderate physical activity, more than 74 min of vigorous physical activity or more than 599 min of metabolic equivalent (MET) per week. Otherwise, the group was defined as inactive [30]. Smoking was categorized as “yes” or “no”: smokers were defined as participants who had smoked 100 cigarettes in their lifetime and who also reported how many cigarettes they smoked per day in the past 30 days. The rest were defined as non-smokers [31].

### 2.3. Statistical Analysis

**Figure 2:**
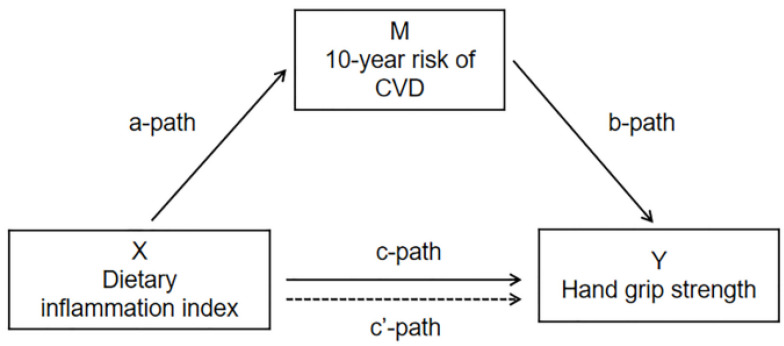
Path diagram of the mediation analysis models.

Continuous variables were described by means and standard errors (SE), and comparisons were made using t-tests for independent samples. Categorical variables were described by unweighted frequencies, weighted percentages and standard errors, and comparisons were made using chi-square tests. Linear regression models were used to explore the relationship between DII, 10-year CVD risk, relative grip strength and the interaction between DII and 10-year CVD risk in different models. To analyze whether 10-year CVD risk mediates between DII and relative grip strength, we used simple mediation models, including Paths A, B and C (shown in Figure 2). The total effect estimated the effect of DII (exposure) on relative grip strength (outcome). Path A assessed the effect of DII and 10-year CVD risk (mediator). Path B evaluated the association between 10-year CVD risk and relative grip strength. Path C (direct effect) provided an estimate of the direct effect of 10-year CVD risk on the association between DII and relative grip strength. The mediated effect was calculated as (mediated effect/total effect) × 100%.

All statistical analyses were performed in IBM SPSS 26.0. Mediation analyses were carried out in IBM SPSS 26.0 using the PROCESS v2 16.3 plug-in. A 2-sided *p* value lower than 0.05 indicates the significance of the results, and *p*-interaction less than 0.1 was considered significant [32].

## 3. Results

### 3.1. Characteristics of Participants

The study participants’ baseline characteristics are presented in Table 1. Participants were categorized as anti-inflammatory diet (*n* = 2846) and pro-inflammatory diet (*n* = 2845). The differences between anti-inflammatory and pro-inflammatory diets were statistically significant (*p* < 0.001) in terms of gender, BMI, race, energy intake, education level, physical activity, smoking status, hypertension medication and marital status. Furthermore, statistically significant differences were also found between participants with relative handgrip strength and different dietary inflammatory index (*p* < 0.001).

Table 2 shows the results of linear regression models of inflammatory diet and 10-year risk of CVD on relative grip strength in different models. In the original model, inflammatory diet and relative grip strength are negatively correlated, and after controlling for covariates (sex, age, race, education level, marital status, physical activity, smoking status and energy intake), we can still find DII and 10-year CVD risk are negatively associated with relative grip strength (Model 3).

### 3.2. Mediation Analysis of 10-Year Risk of CVD

Based on the above analysis, we analyzed the relationship between DII and relative grip strength using the Framingham 10-year CVD risk as a mediating variable. All mediator analyses were performed based on adjustment for gender, age, race, education level, marital status, physical activity, smoking status and energy intake.

As shown in Table 3, DII was positively associated with 10-year risk of CVD (*p* < 0.001) and negatively associated with relative grip strength (*p* < 0.001). In addition, 10-year CVD risk was negatively associated with relative grip strength (*p* < 0.001). It was estimated that 10-year risk of CVD mediated 11.8% of the total association between DII and relative grip strength.

Considering the effect of sex and age, we performed a subgroup analysis of subjects in Table 4. In women, there was a 10.0% mediating effect of 10-year CVD risk between DII and relative grip strength. However, in the male population, we did not find a role for 10-year risk of CVD in the association between inflammatory diet and relative grip strength. Similar results were seen in the subgroup analysis of age groups. The 10-year risk of CVD partially mediated the relationship between DII and relative grip strength in the age group 30–44 years. However, it was not mediated in participants older than or equal to 45 years.

## 4. Discussion

The present study was conducted to analyze the relationship between dietary inflammatory index, 10-year CVD risk and relative handgrip strength and the mediating role played by 10-year CVD risk on the basis of the NHANES. To our knowledge, this is the first investigation of the relationship among them, and the results of our analysis confirm our hypothesis. The main findings of our study are as follows: Firstly, both DII and 10-year CVD risk were negatively associated with relative handgrip strength, and DII was positively associated with 10-year CVD risk. In addition, 10-year CVD risk mediated the effect between DII and relative handgrip strength, with 11.8% of the effect being mediated by 10-year CVD risk. Finally, the mediating role of 10-year CVD risk varies by sex and age. The 10-year CVD risk plays a partially mediated role of 10.0% in females and no mediating role in males. Similarly, a stratified analysis of age found that the relationship between DII and relative grip strength was fully mediated by 10-year CVD risk in the 30–44 age group but not in people 45 years and older.

The intake of pro-inflammatory foods or nutrients can increase levels of inflammation in the body, and inflammation can contribute to the development of CVD events. A study of American men and women found that those on a pro-inflammatory diet had a 38% higher risk of CVD than subjects on an anti-inflammatory diet [8]. Similar results were obtained in our study, meaning that the dietary inflammatory index and 10-year CVD risk are positively correlated. It is now widely accepted that inflammation is one of the underlying mechanisms for the development of chronic diseases such as CVD [33] and that diet is an important factor for inflammation in the body. Therefore, we speculate that diet plays an important role in CVD development. Atherosclerosis is the most important cause of CVD formation [34]. High intake of inflammatory foods accelerates the inflammatory response in the body. Endothelial cells are damaged during inflammation and increase in adhesion, which promotes the expression of adhesion factors that cause leukocytes to attach to the vessel wall and play an important role in the formation of atherosclerosis [35]. The benefits of an anti-inflammatory diet for the control of CVD are reflected in the improvement of endothelial function [36]. Diet is a modifiable and preventable risk factor. Therefore, dietary patterns with less inflammation and strategies to increase the intake of anti-inflammatory foods can provide great value in the prevention of CVD disease.

Our study showed a significant negative correlation between high consumption of a pro-inflammatory diet and relative grip strength. The results of a meta-analysis showed that the risk of death for adults with the lowest grip strength compared to the highest grip strength OR was 1.67 [37]. Dissipation of muscle strength was associated with impaired muscle protein synthesis, imbalance of growth and sex hormones, oxidative stress and increased inflammatory cytokines [38,39]. Previous studies have shown that diet plays a potentially beneficial role in maintaining and improving grip strength through the intake of anti-inflammatory nutrients [40,41]. A study of raw garlic consumption (a healthy anti-inflammatory food consumption) showed a mean difference in grip strength of 1.3 kg and 0.7 kg between the lowest and highest raw garlic consumption in men and women [42]. An anti-inflammatory diet could modulate the cellular inflammatory response by inhibiting the expression of intercellular adhesion molecule-1 [43], whereby inflammatory factors inhibit muscle protein synthesis, muscle growth factor and muscle atrophy protein expression, resulting in accelerated breakdown of muscle proteins and ultimately a decrease in muscle strength [44]. Therefore, we hypothesize that inflammatory low-grade foods act to protect grip strength by inhibiting the decline in muscle strength through the tissue cell inflammatory response.

Patients with CVD are at increased risk of frailty due to reduced physical activity and inadequate blood supply to the body. Low grip strength was mentioned earlier as an objective response to frailty. Studies have shown that people with CVD events such as myocardial infarction, and heart failure are at higher risk of frailty [45]. One review suggested that CVD is a risk factor for frailty and that CVD can accelerate frailty [9]. Another systematic review in the elderly showed a 50–54% risk of frailty in patients with heart failure and severe coronary artery disease [11]. The possible explanation for this phenomenon is that CVD leads to reduced physical activity in patients, and at the same time, inadequate blood supply in patients with CVD also causes frailty, and these two pathways explain the increased likelihood of loss of muscle strength, which increases the risk of frailty. Therefore, low CVD risk is beneficial in preventing low grip strength and even frailty.

Our study showed the 10-year CVD risk was mediated 11.8% by the association between DII and relative grip strength. This result reminds us of the importance of maintaining and improving grip strength by somewhat reducing the 10-year risk of CVD, especially in populations with high dietary inflammation. Interestingly, we found gender and age specificity in the mediating role of 10-year CVD risk between DII and relative grip strength. Gender is thought to be an important factor influencing health conditions [46], and an NIH study supports this view. For example, previous studies have shown that women are more likely to have depression, women consume more pro-inflammatory foods, and men are at higher CVD risk [47,48,49]. For the female population, 10-year risk of CVD can partially mediate the association between DII and relative grip strength, but in the male population, the effect of inflammatory diet on grip strength does not act through future 10-year risk of CVD, which may provide new insights into the maintenance and improvement of grip strength in gender-specific populations. In addition, our age-specific panel analysis also found that 10-year risk of CVD was not mediated in the middle-aged and older age groups. This finding suggests that CVD risk reduction behaviors, such as blood pressure control and smoking cessation, may not be the focus for reducing the effects of inflammatory diet on grip strength weakening in middle-aged and older age groups, in contrast to the partially mediated effect in the 30–44 age group. It is suggested that in younger age groups, reducing the 10-year risk of CVD would be the focus for preventing grip strength weakening

There are certain advantages of our study. Firstly, NHANES was conducted based on rigorous data collection procedures, a complex probability sampling design and a rigorous review that reduced bias and ensured a representative national sample was obtained. Secondly, this is the first study to explore the relationship among DII, 10-year CVD risk, relative handgrip strength and the mediating role of 10-year CVD risk. Of course, we also need to acknowledge certain limitations of our project. First of all, the dietary data were obtained through self-reporting and affected by recall bias and errors of understanding. Furthermore, since this is a cross-sectional study, exact causality cannot be obtained, and the results cannot necessarily be extrapolated; a prospective study with a larger sample is needed for confirmation.

In summary, our study is the first to find that 10-year CVD risk modulates the relationship between DII and relative handgrip strength to some extent and that this mediating effect plays out to varying degrees across gender and age groups. Prospective studies to provide more robust evidence for this relationship are necessary.

## 5. Conclusions

Reducing 10-year CVD risk could attenuate the effect of inflammatory diet on relative grip strength impairment, and in different sex and age groups, the association between DII and relative grip strength is mediated by 10-year CVD risk to varying degrees. Therefore, we recommend reducing the effect of inflammatory diet on grip strength impairment by, for example, lowering blood pressure or quitting smoking (one of the parameters of the FRS), especially for females and non-elderly. Further studies are essential to confirm these results in a prospective design and to explore the mechanisms underlying these associations.

## Figures and Tables

**Table 1:** Characteristics of participants with anti-inflammatory diet and pro-inflammatory diet (Mean (SE)/%(SE)).

Characteristics		Anti-Inflammatory Diet (*n* = 2846)		Pro-Inflammatory Diet (*n* = 2845)		*χ^2^/t*	*p*
		*n*	Mean (SE)/% (SE)	*n*	Mean (SE)/% (SE)		
10-year risk of CVD (%)		—	8.9 (7.4)	—	8.8 (7.5)	−0.12	0.905
Relative handgrip strength (kg/BMI)		—	1.3 (0.4)	—	1.1 (0.4)	−14.29	<0.001
Age (year)		—	51.4 (14.9)	—	51.9 (14.3)	1.33	0.185
BMI (kg/m^2^)		—	28.7 (6.3)	—	29.6 (6.9)	5.47	<0.001
Energy Intake (kcal)		—	2445.0 (809.3)	—	1601.5 (557.5)	−45.78	<0.001
Gender						206.94	<0.001
	Males	1667	59.7 (1.5)	1124	40.3 (1.5)		
	Females	1179	40.3 (1.5)	1721	59.7 (1.5)		
Race						57.28	<0.001
	White	1619	52.3 (1.4)	1478	47.7 (1.4)		
	Black	508	40.6 (2.0)	743	59.4 (2.0)		
	Other	719	53.5 (2.5)	624	46.5 (2.5)		
Education						54.61	<0.001
	<12 years	459	40.2 (2.4)	682	59.8 (2.4)		
	>=12 years	2387	52.5 (1.1)	2163	47.5 (1.1)		
Marital status						62.04	<0.001
	Single	736	44.3 (3.4)	925	55.7 (3.4)		
	Married	1796	54.4 (3.5)	1503	45.6 (3.5)		
	Divorced	314	43.0 (2.0)	417	57.0 (2.0)		
Physical activity						59.79	<0.001
	Inactive	965	47.1 (1.4)	1249	52.9 (1.4)		
	Active	1881	57.7 (1.4)	1596	42.3 (1.4)		
Smoking status						86.72	<0.001
	No	2517	57.1 (1.1)	2258	42.9 (1.1)		
	Yes	329	36.5 (2.7)	587	63.5 (2.7)		
Diabetes						2.03	0.155
	No	2539	54.0 (1.0)	2504	46.0 (1.0)		
	Yes	307	47.3 (3.7)	341	52.7 (3.7)		

Notes: Weighted mean (standard error) for continuous variables and percentage (standard error) for categorical variables.

**Table 2:** Linear regression models of associations between inflammatory diet and 10-year risk of CVD on handgrip strength.

	Model 1			Model 2			Model 3		
	β	95%CI	*p*	β	95%CI	*p*	β	95%CI	*p*
Inflammatory diet (reference = Anti)	−0.160	(−0.190,−0.130)	<0.001	−0.053	(−0.083,−0.023)	0.001	−0.054	(−0.086,−0.022)	0.002
10-year risk of CVD	0.001	(−0.001,0.004)	0.383	−0.009	(−0.011,−0.007)	<0.001	−0.014	(−0.016,−0.012)	<0.001

Model 1 = inflammatory diet + 10-year risk of CVD; Model 2 = Model 1 + age + gender + race + education + marital status; Model 3 = Model 2 + physical activity + energy intake + Smoking status.

**Table 3:** Mediation effect of 10-year risk of CVD on the association between DII and handgrip strength.

Mediator	Sample	Exposure to Mediator	Mediator to Outcome	Direct Effect	Mediated (Indirect Effect)	Total Effect (Exposure to Outcome)	Proportion Mediated (%)
10-year risk of CVD	5691	0.123 (0.037) *p* < 0.001	−0.013 (0.001) *p* < 0.001	−0.015 (0.003) *p* < 0.001	−0.002(0.001) 95%CI (−0.003,−0.001)	−0.017 (0.003) *p* < 0.001	11.8

**Notes:** Exposure: DII; outcome: handgrip strength; model adjusted for age, gender, race, education, marital status, energy intake, physical activity, smoking status.

**Table 4:** Mediation effect of the 10-year risk of CVD on the association between DII and handgrip strength by sex and age group stratification.

	Sample	Exposure to Mediator	Mediator to Outcome	Direct Effect	Mediated (Indirect Effect)	Total Effect (Exposure to Outcome)	Proportion Mediated (%)
**Stratification by Sex**							
Males	2791	0.058 (0.053) *p* = 0.274	−0.015 (0.002) *p* < 0.001	−0.014 (0.004) *p* = 0.002	−0.001(0.001) 95%CI (−0.003, 0.001)	−0.014 (0.004) *p* = 0.001	-
Females	2900	0.138 (0.035) *p* < 0.001	−0.014 (0.002) *p* < 0.001	−0.018 (0.003) *p* < 0.001	−0.002(0.001) 95%CI (−0.003, −0.001)	−0.020 (0.003) *p* < 0.001	10.0
**Stratification by Age group**							
30–44	2070	0.068 (0.033) *p* = 0.038	−0.032 (0.003) *p* < 0.001	−0.016 (0.005) *p* = 0.002	−0.002 (0.001) 95%CI (−0.005, −0.001)	0.018 (0.005) *p* < 0.001	11.1
45–64	2450	−0.087 (0.064) *p* = 0.172	−0.016 (0.001) *p* < 0.001	−0.015 (0.004) *p* < 0.001	0.001(0.001) 95%CI (−0.001,0.003)	−0.014 (0.004) *p* < 0.001	-
≥65	1171	0.187 (0.102) *p* = 0.067	−0.011 (0.002) *p* < 0.001	−0.010 (0.006) *p* = 0.076	−0.002(0.001) 95%CI (−0.005,0.000)	−0.012 (0.006) *p* = 0.035	-

Notes: Exposure: DII; mediator: 10-year risk of CVD; outcome: handgrip strength; model adjusted for age, gender, race, education, marital status, energy intake, physical activity, smoking status.

## Data Availability

Data described in the manuscript, code book, and analytic code will be made publicly and freely available without restriction at [https://www.cdc.gov/nchs/nhanes (accessed on 22 June 2022)].
